# Hunting Down the Chimera of Multiple Disciplinarity in Conservation Science

**DOI:** 10.1111/cobi.12183

**Published:** 2014-01-01

**Authors:** SIMON P POOLEY, J ANDREW MENDELSOHN, E J MILNER-GULLAND

**Affiliations:** * Imperial College Conservation Science, Munro Building, Silwood Park CampusBuckhurst Road, Ascot, Berkshire, SL5 7PY, United Kingdom; † School of History, Queen Mary, University of LondonLondon, E1 4NS, United Kingdom

**Keywords:** conceptual challenges, humanities, interdisciplinary, multidisciplinary, natural sciences, social sciences, transdisciplinary, ciencias naturales

## Abstract

The consensus is that both ecological and social factors are essential dimensions of conservation research and practice. However, much of the literature on multiple disciplinary collaboration focuses on the difficulties of undertaking it. This review of the challenges of conducting multiple disciplinary collaboration offers a framework for thinking about the diversity and complexity of this endeavor. We focused on conceptual challenges, of which 5 main categories emerged: methodological challenges, value judgments, theories of knowledge, disciplinary prejudices, and interdisciplinary communication. The major problems identified in these areas have proved remarkably persistent in the literature surveyed (c.1960–2012). Reasons for these failures to learn from past experience include the pressure to produce positive outcomes and gloss over disagreements, the ephemeral nature of many such projects and resulting lack of institutional memory, and the apparent complexity and incoherence of the endeavor. We suggest that multiple disciplinary collaboration requires conceptual integration among carefully selected multiple disciplinary team members united in investigating a shared problem or question. We outline a 9-point sequence of steps for setting up a successful multiple disciplinary project. This encompasses points on recruitment, involving stakeholders, developing research questions, negotiating power dynamics and hidden values and conceptual differences, explaining and choosing appropriate methods, developing a shared language, facilitating on-going communications, and discussing data integration and project outcomes. Although numerous solutions to the challenges of multiple disciplinary research have been proposed, lessons learned are often lost when projects end or experienced individuals move on. We urge multiple disciplinary teams to capture the challenges recognized, and solutions proposed, by their researchers while projects are in process. A database of well-documented case studies would showcase theories and methods from a variety of disciplines and their interactions, enable better comparative study and evaluation, and provide a useful resource for developing future projects and training multiple disciplinary researchers.

## Abstract (ES)

Cazando la Quimera de la Multidisciplina en la Ciencia de la Conservación

**Resumen:**

El consenso es que tanto los factores ecológicos como los sociales son dimensiones esenciales de la investigación y la práctica de la conservación. Sin embargo, mucha de la literatura sobre colaboraciones multidisciplinarias se enfoca en las dificultades de sobreponerse a ésta. Esta revisión de los retos de conducir una colaboración multidisciplinaria ofrece un marco de trabajo para pensar sobre la diversidad y la complejidad de este esfuerzo. Nos enfocamos en los retos conceptuales, de los cuales emergieron cinco categorías principales: retos metodológicos, juicios de valor, teorías del conocimiento, prejuicios disciplinarios y comunicación interdisciplinaria. Los problemas más grandes que se identificaron en estas áreas han comprobado ser extraordinariamente persistentes en la literatura revisada (c. 1960 – 2012). Las razones por las cuales no se aprende de estas fallas anteriores incluyen la presión para producir resultados positivos y pasar por alto desacuerdos, la naturaleza efímera de muchos proyectos y la resultante falta de memoria institucional, y la aparente complejidad e incoherencia del esfuerzo. Sugerimos que la colaboración multidisciplinaria requiere de integración conceptual entre integrantes de equipo cuidadosamente seleccionados unidos en la investigación de una pregunta o problema compartidos. Resaltamos una secuencia de pasos de nueve puntos para establecer un proyecto multidisciplinario exitoso. Esto incluye puntos sobre reclutamiento, involucramiento de las partes interesadas, desarrollo de preguntas de investigación, negociación de dinámicas de poder, valores escondidos y diferencias conceptuales, explicar y escoger métodos apropiados, desarrollar un lenguaje compartido, facilitar la comunicación continua y discutir la integración de datos y los resultados de los proyectos. Mientras que se han propuesto numerosas soluciones a los retos de la investigación multidisciplinaria, las lecciones aprendidas continuamente se pierden cuando los proyectos terminan o los individuos con experiencia continúan en otros proyectos. Instamos a los equipos multidisciplinarios a capturar los retos reconocidos, y soluciones propuestas, por sus investigadores mientras los proyectos están en proceso. Una base de datos de casos bien documentados podría mostrar teorías y métodos a partir de una variedad de disciplinas y sus interacciones, permitir mejores estudios comparativos y evaluaciones y proporcionar un recurso útil para desarrollar futuros proyectos y entrenar investigadores multidisciplinarios.

## Introduction

For anyone tackling real world environmental problems, the challenges of multiple disciplinary collaboration are virtually unavoidable. Indeed, the pressure of peer expectations and funding requirements make it appear that this is the *sine qua non* for successful and useful research on environmental challenges. Researchers from different disciplines are encouraged to come together to form a kind of chimera, in the biological sense of a single animal composed of several different populations of genetically distinct cells. Despite the assumed virtues of multiple disciplinary collaboration however, much of the literature has focused on the problems (di Castri 1976), barriers (Fox et al. 2006; Adams 2007; Stafford-Smith et al. 2012), obstacles (Endter-Wada et al. 1998; Campbell 2005), difficulties (Lélé & Norgaard 2005), and challenges (Zube 1982; Mascia et al. 2003) of undertaking this kind of research. Some of these authors express these problems so persuasively that multiple disciplinarity can seem an impractical ideal, more akin to the mythical chimera: an ungainly (and impossible) conglomerate of a lion’s head, a goat’s body, and a serpent’s tail.

At first glance, a review of the literature over the last few decades conjures up the hydra: a multitude of different kinds of problems beyond the capacity of anyone to comprehensively resolve. We argue that to cut through this tangle, it is necessary to clarify what is meant by multiple disciplinary collaboration; identify the core recurrent problems of doing it; and devise a coherent approach to overcoming these problems and monitoring research outcomes.

First, some clarification of terminology is necessary. The proliferation of research activities across disciplines over the past half-century has inspired a wealth of classification schemes for multiple disciplinary studies (see Klein [2010] for a summary). We use *multiple disciplinary* to cover multidisciplinary, interdisciplinary, and transdisciplinary approaches. Where differentiated, these terms for multiple disciplinarity are usually defined as follows (adapted from Tress et al. 2006). *Multidisciplinary* projects involve different academic disciplines researching a single problem or theme but working in parallel without integration. *Interdisciplinary* projects involve unrelated academic disciplines in a way that requires them to cross disciplinary boundaries to create new knowledge and theory in pursuit of a common research goal. *Transdisciplinary* projects integrate academic researchers from unrelated disciplines, and nonacademic participants, in pursuing a common goal, and creating new knowledge and theory.

The natural sciences study the physical or natural world (including biology, chemistry, earth science, and physics). The social sciences study human society and behavior (e.g., sociology, psychology, and economics). The humanities study the human condition (e.g., philosophy, literature, and religious studies). Subjects such as history straddle the latter 2 categories.

In a widely cited *Conservation Biology* editorial, Mascia et al. (2003) bemoaned the limited impact of conservation science. They attributed the research–implementation disconnect to a lack of attention to social factors in determining the success or failure of conservation interventions and called on governments and NGOs to bring the social sciences into the mainstream of conservation. Of course, as they observed, the question remained *how* were the social sciences to be integrated, and what were the principal barriers—or more positively, challenges—to effecting this integration (cf Meffe et al. 2006). By reviewing challenges to multiple disciplinary research in conservation, this paper offers a framework for thinking about the sometimes bewildering diversity and complexity of this endeavor.

## Methods

**Figure 1:**
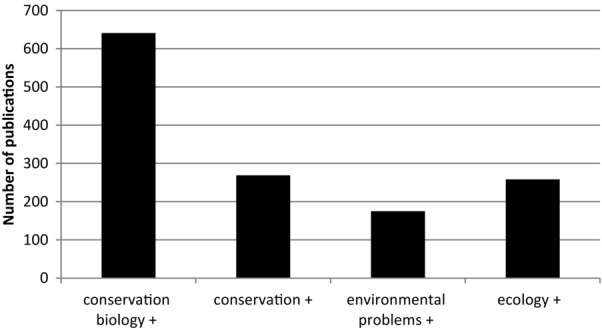
Numbers of publications (published from 1986 to 2010) found using 4 selected search terms paired with “multidisciplinary.”

**Figure 2:**
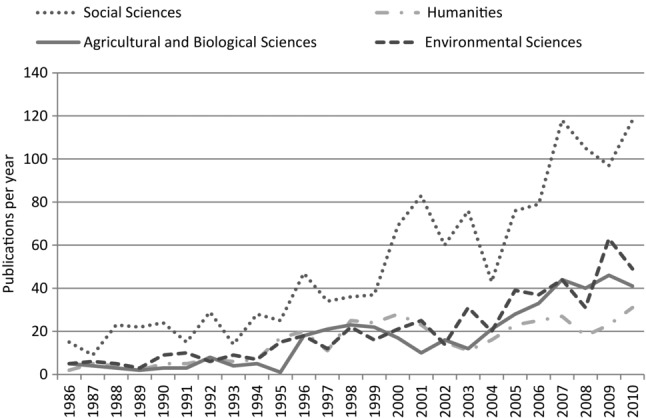
SCOPUS database search results showing number of papers, books, and reviews (1986-2010) described as “multidisciplinary,” by disciplinary grouping.

We used SCOPUS (an abstract and citation database of peer-reviewed literature) and JSTOR (a digital library of peer-reviewed articles) to find the most productive search terms (yielding the most results) to capture articles on multiple disciplinary research on environmental problems (1960–2010). We used the 4 SCOPUS categories which yielded the most results, and added arts and humanities, as a neglected dimension. In JSTOR we used the categories area studies, biological sciences, development studies, social sciences, and humanities. We searched for the terms *conservation biology*, *conservation*, *environmental problem*, and *ecology*—each paired with the term *multidisciplinary* (see Figs.1 & 2). Individual conservation-specific journals including *Conservation Biology*, *Ecology and Society*, *Environmental Conservation*, and *Oryx* were also searched for the term *multidisciplinary*. The term *multidisciplinary* was used because it captured (was also used in) most papers also described as *transdisciplinary* or *interdisciplinary*.

The searches yielded nearly 800 papers from 61 journals, most from 1986 onward. The most productive SCOPUS categories were environmental science, agricultural and biological sciences, social sciences, and earth and planetary sciences. We refined the selection by choosing only papers written in English that explicitly addressed the challenges of multiple disciplinary research on problems of the environment involving natural scientists with social scientists or humanities researchers or that were the outputs of such collaborative research. We sorted these papers into categories by abstract and key words: case studies (45); secondary literature (26); position papers (68); reviews or histories (31); and tools (methods 69 papers, and theories, 40).

These papers were imported into Mendeley reference management software and sorted into categories by type of paper (review, editorial, etc. [http://www.iccs.org.uk/research-themes/past-projects/sharpen/]). S.P. read and coded these using a grounded theory approach, open to emergent themes and theoretical categories (Strauss & Corbin 1998). Codes were devised for disciplines involved; mode of multiple disciplinary work; and keywords and themes as they emerged.

## Results

Across the time period surveyed (with key recent papers added) certain challenges to multiple disciplinary work recurred. These were divided into structural, conceptual, and practical challenges. Structural challenges—educational and sectoral institutional cultures and boundaries and how these are reflected in and entrenched by allocations of funding and policy and managerial responsibilities—are too complex and wide-ranging to include here. They arise from the widely held dualism in Western society between environmental subjects and issues which are coupled with the natural sciences and social and cultural issues which are coupled with the social sciences and humanities (Head et al. 2005; Strang 2009). Practical challenges include recruitment, allocation of resources across disciplines and territories, managing personalities, and so forth. We chose to focus on conceptual challenges. Five main categories emerged (from most-mentioned to least): methodological challenges; value judgments; theories of knowledge; disciplinary prejudices; and interdisciplinary communication. Sixty-eight papers addressing these were reviewed in detail.

### Methodological Challenges

The key methodological challenges which emerged from the papers reviewed were time and phasing; scale; data integration and management; and understanding human motivations and behavior.

Most academic researchers struggle to keep abreast of the literature in their own disciplines, let alone tackle related literatures in other fields (Campbell 2005; Drew & Henne 2006; Welch-Devine & Campbell 2010). A review of articles evaluating interdisciplinary research found virtually no crossover between natural science and social science journals (MacMynowski 2007). This makes it unlikely that researchers will be aware of methodological developments in fields other than their own.

A common theme is the need to spend more time on the initial stages of multiple disciplinary projects. This is important for developing mutual understanding and trust around unfamiliar research methods. This heavy time investment has costs for academic researchers, notably in time away from productive disciplinary research and slowed publication rates. Further, it requires patience and understanding from funders and managers and stakeholders not involved in technical aspects of the research (Cowling et al. 2008).

Regarding phasing, because many natural scientists regard social science as informative and facilitative for policy and implementation processes, ignoring the importance of data collection and analysis in social science research, social science is often consigned to a supportive role to the primary research (Endter-Wada et al. 1998). Social scientists are therefore introduced at a later stage of the project, are not involved in the framing of the project, have insufficient funds, time and baseline data to conduct their research adequately, and arrive too late really to collaborate (Campbell 2005; Strang 2009; Duraiappah & Rogers 2011; Sievanen et al. 2012). However, this gives rise to a circular problem: for while a “problem cannot be defined until a working team is in place… it is impossible to know how deeply to involve specific team members until the problem has been defined” (Nicolson et al. 2002: 380).

Time budgeting for data collection is also a challenge, with major mismatches in timescales required by different disciplines (di Castri 1976; Drew & Henne 2006). Some qualitative data collection and processing requires a long timescale. Strang (2009: 12) argues that “social science research generally requires far more diverse types of data than is commonly encompassed in natural science projects, and … it takes more time to collect and analyse such a wide range of data.”

An early challenge for project planners is how to cover the range of temporal and spatial scales required to incorporate the social and ecological dimensions of the problem in hand (a persistent challenge noted from di Castri et al. 1981; Scoones 1999; Gibson et al. 2000; through to Collins et al. 2011). Heemskerk et al. (2003: 9) report a mismatch between “the spatial and temporal scales at which ecosystems function and ecological problems manifest … [and] the scale at which management and research occur.” Investigating the interaction of processes occurring at different temporal and spatial scales requires careful thought about which data should be collected and at which scales. Observations of long-term environmental and behavioral changes (where experimental methods may not be feasible) may require very long-term data, or in the case of research, very long-lasting projects (Collins et al. 2011).

The perception persists that natural scientists favor quantitative data over qualitative data because it is easier to compare and generalize from and is perceived as more objective—as opposed to social scientists and humanities researchers who favor qualitative data (Heemskerk et al. 2003; Drew & Henne 2006). However, some authors question this alleged dualism of preference, recognizing that it can “be a source of tension between scientists within any scientific field” (Heemskerk et al. 2003: 3). Social scientists of different kinds may favor predictive or understanding theories, for instance, and such preferences affect the kinds of data they collect and the methods they use. Comments on the difficulties of integrating natural science and social science data often actually refer to difficulties in integrating quantitative and qualitative data.

Hadorn et al. (2010) outline 5 classes of integration methods, 3 of which predominate in the reviewed literature: dialogue methods; model-based methods; and common-metric-based methods. Models are a particularly popular solution for integrating, developing, and managing data and conceptual frameworks (e.g., Zube 1982; Heemskerk et al. 2003; Milner-Gulland 2012; Schlüter et al. 2012). For instance, household utility models are used to understand how individual households allocate labor for natural resource use. However if models have inscrutable inner workings, then collaborating researchers must take the outputs of these models on faith (Nicolson et al. 2002). This is a particular challenge in multiple disciplinary work involving researchers with no background in modeling. Those who do not understand models tend not to engage with their workings or outputs. They distrust counter-intuitive model outputs rather than being motivated to investigate the intermediate relationships that generated these results (Nicolson et al. 2002).

Strang (2009) argues that the systems theory underlying much of the modeling of social–ecological systems aims to maintain a manageable multiplicity of variables and render each factor transparent, definable, and homogenized. This makes such modeling unsuited to “expressing … complex and intangible realities of power relations, belief systems, values, understandings of environmental processes, affective responses to place, identity, social relations and so forth” (Strang 2009: 14). The subtlety of the interrelations of the material and social worlds is distorted and explanatory ethnographic context is omitted, rendering human behavior a black box. Qualitative data tend to be compressed “into extinction” (Strang 2009: 14).

It is challenging to model the feedbacks between human and ecological systems, particularly to incorporate influences on human behavior external to ecological feedbacks (Collins et al. 2011). Although policy makers are clamoring for research quantifying the impacts of humans on biological systems or the effects of biodiversity loss on human well-being, we need more research on feedbacks between changes to human well-being and human behavior toward nature (Miller et al. 2012). Conservation interventions based on statistical models may themselves change conditions, thereby invalidating the analysis upon which they were predicated (Milner-Gulland 2012). To overcome this requires a better understanding of how individual humans, and communities, make decisions in a changing world.

“Most of the problems of conservation are to do with people,” comments Adams (2007). It is thus surprising that St John et al. (2010) have to chide ecologists and conservationists for not using tried-and-tested methods employed by social psychologists to explore (and thus, hopefully, change) individuals’ motivations for specific problematic behavior (see also Saunders et al. 2006). Most conservation research uses economic models assuming people behave rationally, ignoring the importance of key determinants of individual behavior identified by social psychologists, such as subjective and descriptive norms and moral obligations (St John et al. 2010). Jones et al. (2011) advocate “mental models” for understanding individual behavior.

Understanding human motivations and behavior requires more than engaging with just the cognitive, behavioral, and physiological dimensions of our interactions with the environment. It requires an engagement with culture(s). Within Europe, Ressurreição et al. (2012) found local cultural values significantly influence willingness to pay for the conservation of particular marine species. The ecosystem services literature was developed primarily by western ecologists and economists focused on capitalist values and systems, where economics plays a central role in assessing values and shaping societies (Reyers et al. 2010). This sidelines the values espoused by other philosophies (Sessions 1995) and cultures.

Head et al. (2005) attack the popular misconception that science and culture are distinct entities, arguing that culture pervades all of our lives and institutions. They recommend more qualitative research on the (dynamic) cultural conceptions of the environment of all participants and stakeholders, as well as the cultures of disciplines and how they frame researchers’ world views. Ludwig et al. (2001: 503) observe that “environmental problems may reflect our own culture and attitudes as much as a scientific or technical problem” (and see Cronon 1995). Science and technology studies (STS) and public understanding of science (PUS) are making significant contributions to research in this area. Some radical approaches are emerging—for instance, rethinking the roles of scientific experts and the public and hybridizing rather than trying to separate science and politics (Lane et al. 2011).

### Normative Concerns and Priorities

Conservation scientists are explicit about the “normative postulates” which form “the basis of an ethic of appropriate attitudes toward other forms of life” (Soulé 1985: 730). Defining what he called, more narrowly, conservation biology, Soulé maintained that “ethical norms are a genuine part of conservation biology, as they are in all mission- or crisis-oriented disciplines” (Soulé 1985: 727). The centrality of an underlying ethics to conservation science has been asserted many times (e.g., Noss 1999). However, these values are often articulated as “conservation principles,” which express scientific assumptions and shift the focus to mechanisms for implementation rather than ethical questions (Ruitenbeek 1997).

In this context, the word *conservation* implies action and a stance. However, when it becomes *conservation science* this is problematic because for many being a scientist implies a need for detached objectivity (Wilhere 2011). There is a longstanding tension in most natural and some social sciences between scientific objectivity and social engagement. Ecologists and conservation scientists are anxious for their work to appear value free and many distance themselves from environmental advocacy—while promoting the social relevance of their work (Nelson & Vucetich 2009; Wilhere 2011).

Lélé and Norgaard (2005) observe that most natural scientists believe that science is value neutral but argue that seeking objective measures such as ecosystem integrity or green GDP does not eliminate value judgments—each is shaped by the choice of ultimate values or relevant variables or decisions concerning the aggregation of disparate values. Such hidden value judgments can mislead those unaware of the value-laden nature of these apparently objective analyses and cause friction in multiple disciplinary research teams when social scientists point them out (Lélé & Norgaard 2005).

Sandbrook et al. (2013) distinguish between 2 kinds of social researchers: those aiming to contribute to the mission of conserving biodiversity (e.g., West et al. 2006; Büscher 2013) and those studying conservation as a social phenomenon without necessarily sharing its mission. Sandbrook et al. urge conservationists not to reject the findings of the latter because they may not be on mission or may be hostile (e.g., Guha 1997). Engaging offers the opportunity for critical self-reflection and facilitates an appreciation of the larger context in which conservation interventions are performed and experienced.

Much conservation science and sustainable development research is informed by notions of what good outcomes are. However, Chan et al. (2007) note that less attention is paid to the ethical implications of the research process. Minteer and Collins (2005: 1804) maintain that although environmental ethics (Pojman 2011) is good on conceptual issues, “there is … no … subfield of applied or practical ethics devoted expressly to investigating the special kind of ethical issues raised within ecological research and biodiversity management contexts.” In light of an increased profile for market-based conservation, there are concerns that social scientists researching the social impacts of conservation interventions may be sidelined (Welch-Devine & Campbell 2010). Nearly a quarter of a century after Soulé’s statement on ethics and conservation, the training many conservationists receive (mostly natural sciences) still does not incorporate applied ethics (Drew & Henne 2006; Newing 2010).

### Theories of Knowledge

Theories of knowledge consider what we think we can know and how we can know it. The importance of resolving conceptual differences in the initial stages of interdisciplinary research projects is emphasized by many authors (e.g., Nicolson et al. 2002; Heemskerk et al. 2003; Campbell 2005; Lélé & Norgaard 2005; Newell et al. 2005; Dewulf et al. 2007; Khagram et al. 2010; Sievanen et al. 2012). The perception that this breaks down along subjective versus objective, qualitative versus quantitative, subjectivist versus positivist, social sciences and humanities versus natural sciences lines was identified as widely held (Drew & Henne 2006; Adams 2007; Evely et al. 2010) but too simplistic (Lélé & Norgaard 2005; Khagram et al. 2010). Most of these authors are concerned with either investigating or proposing conceptual frameworks, with the aims of making explicit, defining, and discussing the philosophies of knowledge, theories, and research styles researchers from diverse disciplinary backgrounds bring to framing multiple disciplinary projects. These are usually approaches to the processes of differentiation, clarification, and synthesis required to sort out conceptual differences, rather than discussions of particular theories or concepts (for the latter, see Moran 2010).

### Disciplinary Prejudices

One reason the acknowledgement and resolution of conceptual differences and related choices regarding problem definition, theory choice, and methodology is so difficult is that the multiple disciplinary landscape is structured by often unacknowledged but widely accepted hierarchies of power. Despite calls to integrate the social sciences into research and management of social–environmental systems, scientific institutions and natural scientists retain their preeminent social authority as mediators of truth and knowledge on environmental matters (MacMynowski 2007; Strang 2009; Welch-Devine & Campbell 2010; Sievanen et al. 2012).

MacMynowski (2007) advocates confronting “the exercise of differential power by social and natural scientists, within academia and beyond,” especially regarding debates over subjectivity in research. She argues that “[a] deep normative current persists that valorizes mathematics and physics as the objective scientific ideal and views other research, particularly in the social sciences, to be trailing behind in the quest for rigor and valid knowledge” (MacMynowski 2007: 5). Social sciences which acknowledge the subjectivity of the observer face a power deficit in multiple disciplinary projects.

Most natural scientists “share fundamental, positivist assumptions about the law-like nature of the systems that they study and the search for universal principles of explanation” (MacMynowski 2007: 6). They tend to avoid questions about subjectivity and the role of the researcher, thus maintaining their authority in knowledge production (alongside economists within the social sciences). Such asymmetrical power relationships influence which projects are undertaken, which disciplines are involved and at which stage, conflict resolution, and the level of acceptability of the research outputs to the scientific community (Campbell 2005; Drew & Henne 2006; Sievanen et al. 2012).

A particular complaint of social scientists, borne out by surveys of natural scientists working on multiple disciplinary projects (Fox et al. 2006; Lowe et al. 2009; Welch-Devine & Campbell 2010; Sievanen et al. 2012), is that the latter regard the social sciences instrumentally as a means to communicate their findings to or change the behavior of their target audiences (Whyte 1982; Endter-Wada et al. 1998). Social scientists are thus often brought in at later stages of projects and excluded from the planning process.

This attitude results in natural scientists undertaking tasks such as designing questionnaires and conducting surveys without being aware of the protocols and best practices developed for such work by the social sciences. If this yields poor quality data which cannot generate robust and generalizable results, then this is viewed as a failure of social science techniques, rather than the result of their poor implementation. (Of course, many social scientists do not aim to deliver generalizable results, for example, cultural relativists among anthropologists.) Although natural scientists often assume they can manage the social science work required by their conservation projects, the equivalent assumption that social scientists can do natural science research is seldom heard—in fact the opposite view prevails (Welch-Devine & Campbell 2010).

In an online survey of views on the role of the social sciences in conservation, Fox et al. (2006) found that social scientists felt unvalued by conservation scientists. A survey of social science and humanities scholars on engagement in global environmental change (GEC) research conducted by the International Human Dimensions Programme (IHDP) found that 42% (of 152) agreed that “social/human dimensions of GEC research are not regarded as important or relevant by others in the GEC field” (Duraiappah & Rogers 2011: 24). Lowe et al. (2009) found that most of the ecologists working on the Rural Economy and Land Use (RELU) project who responded to their questionnaire preferred tackling the social dimensions of their work by engaging with stakeholders, rather than with social scientists. Welch-Devine and Campbell (2010) found that some natural scientists felt they were doing social science just by working with local communities. On the other hand, Strang (2009) points out that social scientists should not just wait to be invited onto projects initiated by natural scientists, but should invite natural scientists to collaborate on their projects too.

### Interdisciplinary Communication

Building trust and familiarity with one another’s mental frameworks, including how words and concepts are used by researchers (and stakeholders) from different backgrounds, is judged essential for collaborative multiple disciplinary work (Nicolson et al. 2002; Heemskerk et al. 2003; Dewulf et al. 2007). Project planners are urged to budget for communication-rich face-to-face meetings and time for social interaction (Daily & Ehrlich 1999) and institutions to jointly house collaborating researchers from different disciplines (Heberlein 1988).

Reading the literatures of other disciplines facilitates collaboration across disciplines. It eases the pressure on multiple disciplinary project leaders to familiarize researchers with the terminologies and writing conventions of other disciplines. Unfortunately, few read across disciplines (MacMynowski 2007). Publishing research in the journals of other disciplines also helps break down disciplinary barriers (Wear 1999; Campbell 2005; Drew & Henne 2006). However, the orientation of academic assessment to discipline-specific journals and theoretical work and the perceived lower status of interdisciplinary and applied journals inhibit researchers from publishing multiple disciplinary work (Daily & Ehrlich 1999; Fox et al. 2006; Welch-Devine & Campbell 2010). There are also perceived prejudices against publishing with researchers from other disciplines. Social scientists struggle with the bias toward natural science approaches and methods of conservation journal reviewers and reviewers’ assumption that they are competent to comment on the validity of social science methods (Pickett 1999; Campbell 2005). Equally, a lack of social science reviewers risks allowing the publication of substandard social science.

## Discussion

The prevailing wisdom is that solving environmental problems requires multiple disciplinary research, despite the manifold challenges of enacting it. It is worth recalling that it is not proven that such studies are more successful in delivering new knowledge, or conservation outcomes, than single disciplinary approaches. One factor complicating the assessment of such programs is the pressure exerted by funding bodies to present projects as multiple disciplinary, even if in practice they were not (di Castri & Hadley 1986; Pickett 1999; Huutoniemi 2010). If we are to advocate multiple disciplinary research, it seems necessary to clarify what we mean by this and outline an approach for successfully undertaking such projects. If this is achieved at the conceptual level, structural and practical challenges should be easier to resolve.

Multiple disciplinary collaboration requires conceptual integration among carefully selected multiple disciplinary team members united in investigating a shared problem or question. This integration requires an investment of time at the beginning of the project, which is often hard to achieve given pressure for large projects of limited time span to get underway, show results, and generate outputs. Of course, someone has to have had the initial idea. Ideally they recognize the need for a multiple disciplinary approach and send out feelers to colleagues about the putative nature of such collaboration. We propose it may be preferable to formalize this process for projects not being undertaken by established multiple disciplinary teams. That is, build in a structural requirement for large multiple disciplinary grants to be preceded by a one-year development period (a scoping grant). Prospective participants could develop working relationships and build the conceptual framework together. An anonymous questionnaire survey could help funders and researchers assess the level to which this was achieved and give advance notice of potential problems.

Based on the reviewed literature, we propose that once there is an outline (broad, guiding) question, the following steps should be followed in setting up a successful multiple disciplinary project: (1) recruit the right people (leaders, collaborative workers) from the right disciplines (Nicolson et al. 2002; Sievanen et al. 2012); (2) establish relationships with stakeholders (Drew & Henne 2006); (3) refine the research question and aims with everyone’s input (Heemskerk et al. 2003; Sievanen et al. 2012); (4) develop mutual trust and respect and clarify power relations (Dewulf et al. 2007; MacMynowski 2007); (5) identify, reflect on, and resolve how to accommodate hidden values and conceptual differences (Heemskerk et al. 2003; Lélé & Norgaard 2005; Newell et al. 2005); (6) educate all researchers about the purpose and use of theories, concepts, methods and data from a range of disciplines (Nicolson et al. 2002; Strang 2009); (7) select appropriate methods, jettison superfluous ones, and devise new ones (Pickett 1999; Khagram et al. 2010); (8) discuss data integration and project outcomes (practical and academic; Strang 2009; Sievanen et al. 2012); and (9) develop a shared language and ongoing facilities and opportunities for good communication (Daily & Ehrlich 1999; Newell et al. 2005). The reviewed literature provides useful insights on most of these 9 points, discussed in sequence below.

According to Nicolson et al. (2002: 378), the “right people” are those “committed to studying a complex system by focusing … on the interrelationships among components.” Although researchers should be expert in their own disciplines, “the best disciplinary minds are not necessarily the best interdisciplinary team members.” This is especially true for project leaders. They must be secure enough to explore linkages, simplify and communicate their discipline to others, and guess at the unknown. They must be good listeners whose interest in the problem outweighs the need for power plays and career considerations. In addition, a professional project manager not from any of the disciplines involved could play a key role as a neutral coordinator and facilitator (Heberlein 1988).

Many (e.g., Torkar & McGregor 2012) advocate a community-based conservation approach which requires the input of local stakeholders throughout and at every level. This suggests researchers should engage with traditional, and “popular,” ecological knowledge about specific environments, as well as a spectrum of nonutilitarian values. In practice, these must to be engaged with alongside, rather than subservient to, scientific knowledge and utilitarian and anthropocentric value systems (Fernandez-Gimenez et al. 2007; Shakeroff & Campbell 2007; Pretty et al. 2009; Lane et al. 2011).

If multiple disciplinary conservation research aims to understand and change destructive human behavior, the omission of humanities disciplines producing nuanced cultural analyses is inexplicable (Balsamo & Mitcham 2010). Commentators on the cultural biases and limitations of utilitarian and anthropocentric value systems seldom cite the growing body of research in the ecological humanities which transcends conceptions of natural resources for human use to consider the duty of care to the nonhuman world (Fischer et al. 2007; Robin & Steffen 2007). Jepson and Canney (2003) urge conservationists to re-engage with the ethical and aesthetic arguments which inspire much public interest in conservationism. The outcomes of the applied sciences are interwoven with many factors beyond the control (and focus) of researchers and practitioners. Integrated environmental histories could more adequately frame and interpret the interactions of these interlocking social–ecological systems over time, as has occasionally been suggested (e.g., Worster 1996; Endter-Wada et al. 1998; Szabó 2010) but seldom enacted in multiple disciplinary contexts.

Despite the recognition that cultural and social values and their political expressions drive policy and management priorities, which in turn drive how resources are managed and ultimately how knowledge acquisition is structured and funded to enable this, attempts to engage with the humanities disciplines which study values, ethics, history, and philosophy have not entered the mainstream of ecology or conservation science (Fischer et al. 2007; Reyers et al. 2010; Pooley 2013).

Ideally, all researchers should consider the shaping effects of their personal and disciplinary values, motivations, and conceptual frameworks before they attempt collaborative research. In practice, self-reflection is difficult to achieve, and a variety of proposals are made for tackling this within multiple disciplinary teams (e.g., Newell et al. 2005; MacMynowski 2007; Khagram et al. 2010). Doing so can reveal assumptions about power relations and conceptual frameworks within the group context, allowing these to be renegotiated within the framework of a project.

Whereas project outcomes are straightforward for multiple disciplinary teams in industry, or single disciplinary projects, in multiple disciplinary conservation projects participants usually have a diversity of desired outcomes. Therefore, principal outcomes must be agreed at the outset. It is helpful to address perceptions about the lower status (and quality) of interdisciplinary research. These are largely anecdotal and have been partially challenged by Hicks et al. (2010). They found that more established, larger disciplines are not less inclined to support interdisciplinary research than newer disciplines. Further, interdisciplinary research achieved a higher impact factor than more discipline-specific research. Reyers et al. (2010: 508) note “the large number of inter- and transdisciplinary publications, reviews, special issues and journals available” and question the “frequently expressed concern that disciplinary journals are usually more prestigious and have higher impact factors than interdisciplinary ones.” That said, multiple disciplinary journals require reviewers competent in the major disciplines involved and alert to disciplinary biases (Daily & Ehrlich 1999).

Many, perhaps even most, multiple disciplinary projects are ephemeral. They are also bound up in ways not adequately addressed with the career trajectories of influential individuals and the challenges of assembling and maintaining teams of people with very different personal styles, capabilities, and career aspirations (Zube 1982; Broto et al. 2009). For these reasons, there have been few enduring banks of institutional memory associated with multiple disciplinary research. The experiences gained during projects tend to be lost when projects are wound up, teams fragment, and experienced individuals move on. Multiple disciplinary teams should attempt to capture the challenges recognized, and solutions proposed by, their researchers while projects are in progress (e.g., Lowe et al. 2009; Phillipson et al. 2011). Achieving standardization in definitions of multiple disciplinarity, and techniques and tools of data gathering, would further enable comparative studies (Evely et al. 2010; Newing 2010).

These challenges and recommendations have been gleaned from a long-term review of the literature. What is surprising is the periodic return of calls for major multiple disciplinary programs to tackle social–environmental problems, accompanied by assertions of novelty and calls for new kinds of researchers and research (see UNESCO 1971; Soulé 1985; Folke 2006; Leemans et al. 2009; Stafford-Smith et al. 2012). A productive area for further research and reflection is just why these calls, and many of the challenges they are (repeatedly) required to address have persisted for at least half a century. This is despite some clear advances in the sciences and technologies involved and the many reviews and opinion pieces about how best to carry out multiple disciplinary research.

It may be that we live in an age of multiple disciplinarity now and newer initiatives will provide lasting institutional bases for the accumulation of knowledge about the process of conducting multiple disciplinary research. However, the pressure on the leaders of big funded multiple disciplinary projects to produce positive outcomes will continue to militate against open reflection on the disagreements and failures encountered in such projects. As recommended above, making trial periods and assessment a structural requirement of large-scale project funding may help address this.

The existence of new multiple disciplinary initiatives and institutions should not obscure the considerable but fragmented database that already exists and attempts to capture and analyze it. The accumulation of well-documented case studies on the processes and outcomes of multiple disciplinary research projects should be an important research goal in itself. Captured in an open-access bibliographic database, these would be valuable for training multiple disciplinary researchers. It would showcase theories and methods from a range of disciplines and their interactions (Mascia et al. 2003; Newing 2010). This itself is a recurring recommendation (see Heberlein 1988).

The major categories of challenges to multiple disciplinary research identified in this review should be focal areas for attempts to better facilitate multiple disciplinary research.

We believe the key is to address conceptual challenges early. We have outlined an approach for doing so. Strategies must be monitored and assessed. Apparent obstacles should be periodically assessed against contemporary practices and circumstances so that anecdotal and outdated perceived obstacles (e.g., overwhelming publication bias against multiple disciplinary research) can be dispensed with. This way we may productively pursue chimeras actual, rather than mythical.
